# The ovarian response prediction index (ORPI) as a clinical internal quality control to prevent ovarian hyperstimualtion syndrome

**DOI:** 10.5935/1518-0557.20160021

**Published:** 2016

**Authors:** Joao Batista A Oliveira, Jose G Franco Jr

**Affiliations:** 1 Center for Human Reproduction Prof. Franco Jr.

**Keywords:** Age, AMH, Antral follicle, Ovarian stimulation, ovarian hyperstimulation syndrome, ORPI

The laboratory procedures performed within the context of assisted reproduction are supported by rigorous internal (IQS) and external quality systems (EQS). European directives and recommendations require the use of IQS and EQS and the adoption of quality control principles in areas such as organization, management, personnel, equipment and materials, documentation, record keeping, and quality reviews (The ESHRE Guideline Group on Good Practice in IVF Labs, 2015).

Despite the growing concern with the standardization of laboratory work, little has been done to establish clinical internal quality control measures to systematize and increase the consistency of ovarian stimulation (OS) protocols. The prevention of ovarian hyperstimulation syndrome (OHSS) is a key factor in the safe use of assisted reproduction technologies (ART). Thus, the determination of the ovarian reserve is a mandatory step in the individualization of the dosages of the drugs administered to patients undergoing ART.

The first indicator of a patient's ovarian reserve is her age. Although the number and quality of oocytes decrease with age, the reproductive potential varies dramatically among women within the same age group. Therefore, they might exhibit different responses to OS. Consequently, an individual's chronological age may not be a valuable predictor of fertility, as the latter relates to a biological age defined by hormonal and functional factors. In addition to dynamic tests, several clinical, endocrine, and ultrasound markers have been proposed to predict ovarian response to stimulation. Two of these markers are of particular interest: anti-Müllerian hormone (AMH) and antral follicle count (AFC). The AFC - the number of follicles measuring 2-9 mm in diameter seen in both ovaries on transvaginal ultrasound - has been used to predict the ovarian reserve and patient response to OS. However, the criteria used to categorize antral follicles vary significantly in the literature. AMH, a member of the transforming growth factor beta superfamily, is produced only by the granulosa cells surrounding the pre-antral and small antral follicles. Additionally, AMH is independent of follicle-stimulating hormone (FSH), whereby its levels are a direct measure of the follicular production pool. The serum levels of AMH decrease throughout reproductive life and are undetectable in the postmenopausal period. Recently introduced tests (Gen II Elisa/pre-mixing samples with assay buffer) have increased the stability of AMH dosage results (Craciunas *et al*., 2015).

However, despite the individual predictive power of each marker of ovarian response, the estimations they provide are not error-free. In fact, none of these parameters may be considered to be completely reliable predictors of the number/quality of the remaining oocytes in the ovary, or of the probability of having a successful pregnancy following infertility treatment. Therefore, the prediction of ovarian response based on a single biomarker may not be sufficient to justify the formulation of a treatment plan.

In 2012, our group (Oliveira *et al.,* 2012; 2013) described an ovarian response prediction index (ORPI). The ORPI values were calculated by multiplying the AMH (ng/ml) level by the number of antral follicles (2-9 mm), and then by dividing the outcome by the patient's age (in years). This definition of the ORPI was based on previous evaluations in which ovarian response to stimulation was positively correlated with AMH levels and the AFC, and negatively correlated with patient age. The derivation of the ORPI was intuitive and based on the observed correlations and the testing of different combinations. We sought to propose a simple, easy-to-use index and combined a small number of variables whose associations potentiate the prediction of ovarian response to stimulation in each individual, while compensating for possible individual deficiencies.

The ORPI is a simple three-variable index that offers excellent ovarian response prediction (area under the ROC curve of 0.91), and good predictions for the possibility of collecting > 4 MII oocytes (AUC: 0.84) and excessive ovarian response (AUC: 0.89) in infertile women. The ORPI may be used to improve the cost-effectiveness of ovarian stimulation regimens by guiding the selection of medications and tailoring the dosages and regimens to the current needs of patients.

Four years ago the cutoffs for poor, normal, and excessive ovarian response were adopted at our center along with the recommended dosages for patients in each of the ovarian response categories. The most striking finding was the disappearance of cases of OHSS, especially in patients with an ORPI ≥1.7 at risk of producing ≥15 oocytes offered a preventive protocol with doses of FSH starting at ≤ 112.5 IU and GnRH antagonists to block endogenous discharges of LH. This ORPI value (≥1.7) is our benchmark that indicates high risk for OHSS.

When analyzed properly, the ORPI may help eliminate cases of OHSS and standardize the FSH doses prescribed by the various physicians working at a fertility center, while facilitating the implementation of internal quality control measures in ovarian stimulation protocols.

One of the limitations of the ORPI revolves around the need for each service to establish their own cutoff thresholds for poor, normal, and excessive ovarian response. This effort is strongly based on the proper measurement of AMH levels (modified Beckman-Coulter Gen II ELISA, Elecsys^®^ AMH Cobas) and rigorous ultrasound assessment of antral follicles measuring 2-9 mm.

The points mentioned above encourage the idea that it is possible for IVF units to develop clinical internal quality control protocols for ovarian stimulation procedures.
